# Do retreatment tuberculosis patients need special treatment response follow-up beyond the standard regimen? Finding of five-year retrospective study in pastoralist setting

**DOI:** 10.1186/s12879-017-2882-y

**Published:** 2017-12-12

**Authors:** Fentabil Getnet, Henok Sileshi, Wubareg Seifu, Selam Yirga, Abere Shiferaw Alemu

**Affiliations:** 1 Department of Public Health, College of Health Sciences, Jigjiga University, Jigjiga, Ethiopia; 2 Department of Medical Microbiology, School of Medicine, Jigjiga University, Jigjiga, Ethiopia; 3 Dagu Consulting & Services, Addis Ababa, Ethiopia; 4 Department of Medical Laboratory Science, College of Health and Medical Sciences, Haramaya University, Harar, Ethiopia

**Keywords:** Tuberculosis, Treatment outcome, Retreatment, Dots, Pastoralist

## Abstract

**Background:**

Treatment outcomes serve as proxy measures of the quality of tuberculosis treatment provided by the health care system, and it is essential to evaluate the effectiveness of Directly Observed Therapy-Short course program in controlling the disease, and reducing treatment failure, default and death. Hence, we evaluated tuberculosis treatment success rate, its trends and predictors of unsuccessful treatment outcome in Ethiopian Somali region where 85% of its population is pastoralist.

**Methods:**

A retrospective review of 5 years data (September 2009 to August 2014) was conducted to evaluate the treatment outcome of 1378 randomly selected tuberculosis patients treated in Kharamara, Dege-habour and Gode hospitals. We extracted data on socio-demographics, HIV Sero-status, tuberculosis type, treatment outcome and year using clinical chart abstraction sheet. Tuberculosis treatment outcomes were categorized into successful (cured and/or completed) and unsuccessful (died/failed/default) according to the national tuberculosis guideline. Data was entered using EpiData 3.1 and analyzed using SPSS 20. Chi-square (χ^2^) test and logistic regression model were used to reveal the predictors of unsuccessful treatment outcome at *P* ≤ 0.05 significance level.

**Result:**

The majority of participants was male (59.1%), pulmonary smear negative (49.2%) and new cases (90.6%). The median age was 26 years [IQR: 18–40] and HIV co-infection rate was 4.6%. The overall treatment success rate was 86.8% [95%CI: 84.9% - 88.5%]; however, 4.8%, 7.6% and 0.7% of patients died, defaulted and failed to cure respectively. It fluctuated across the years and ranged from 76.9% to 94% [*p* < 0.001]. The odds of death/failure [AOR = 2.4; 95%CI = 1.4–3.9] and pulmonary smear positivity [AOR = 2.3; 95%CI = 1.6–3.5] were considerably higher among retreatment patients compared to new counterparts. Unsuccessful treatment outcome was significantly higher in less urbanized hospitals [*p* < 0.001]. Treatment success rate had insignificant difference between age groups, genders, tuberculosis types and HIV status (*P* > 0.05).

**Conclusion:**

This study revealed that the overall tuberculosis treatment success rate has realized the global target for 2011–2015. However, it does not guarantee its continuity as adverse treatment outcomes might unpredictably occur anytime and anywhere. Therefore, continual effort to effectively execute DOTS should be strengthened and special follow-up mechanism should be in place to monitor treatment response of retreatment cases.

## Background

Tuberculosis (TB) remains a major global public health threat. It caused an estimated 9.6 million ill cases and 1.5 million deaths globally in 2014. Sub-Saharan Africa suffers the highest rate of cases and deaths relative to population. Ethiopia was the 9th and the 2nd TB high burden country in the globe and Africa respectively by a total of 119,592 notified cases in 2014, and one of the three global multi-drug resistant TB burden countries [1]. The prevalence is even higher in pastoralist regions of Ethiopia [2] that comprise our study site.

Early diagnosis and proper treatment using combined drugs have been the main strategies to control TB and reduce its transmission to others [3]. The treatment regimen is extended for 6 to 8 months for any form of TB including pulmonary smear positive (PTB+), pulmonary smear negative (PTB-) and Extra-pulmonary TB (EPTB) in Ethiopia depending on patient category. New patients are treated with Rifampicin (R), Isoniazid (H), Pyrazinamide (Z) and Ethambutol (E) for 2 months (intensive phase) and RH for 4 months (continuation phase) whereas retreatment cases are treated with RHZES (S, streptomycin) for 2 months, RHZE for 1 month and RHE for the remaining 5 months [4].

To improve treatment adherence and cure rates, Directly Observed Therapy-Short course (DOTS) has been implemented as the standard of care [5]. Patients are observed swallowing each dose of TB medications in front of healthcare providers for the first 2 months and care attendant at home for the remaining months of treatment [4]. Ethiopia has adopted the DOTS strategy since 1992 after successful pilot with the first combined treatment in Arsi and Bale zones, Oromia Region [6] and expanded to primary health care units afterwards [4, 7]. However, TB services that are deep-rooted to complex patient, care provider and health systems related factors are still underutilized globally [8, 9]. Poor compliance to DOTS, either non-adherence or default to the standard treatment and care, is one of the common challenges of DOTS implementation that could result in treatment failure, relapse, death, acquired drug resistance, and prolonged infectiousness of patients [10, 11].

The updated TB Global Plan (2011–2015) had planned to achieve a treatment success rate of 87% in all countries by 2015 [12]. However, it was not achieved by all high burden countries till 2013, and countries like Russia and Brazil carried on the lowest treatment success rates of 68% and 72% by 2013, respectively [1]. Nation or facility specific studies also demonstrated variation across different geographical and political settings [13]. Undesirable TB treatment outcomes could be as troubling as one-third of patients had died or defaulted in Malaysia [14], and 16% and 19% of HIV co-infected smear positive patients defaulted and died respectively in India [15]. It could even be unexpectedly worse in low burden countries; for instance, 23.7% of smear positive patients died in Japan [16]. The problem has been inevitably high in Sub-Saharan countries too; unsuccessful outcome was as high as 30.4% among children in Kinshasa in 2015 [17] and 24.5% in Nigeria in 2012 where 10.9% of patients died [18].

Ethiopia achieved a success rate of 89% for all forms of TB in 2013 [1] and it is supported by facility based studies from various locale of the country [19–24]. However, it has never been consistent throughout the country. The worst treatment outcomes of 31.5% default and 17.4% death rates were reported in 2008 from Gondar Teaching hospital [25] and unsuccessful outcome was even remained at 19.5% 5 years later in 2013 in same town [26]. Similarly, 19.2% of TB patients across the last 5 years had unsuccessful outcome in Bahir Dar [27].

This highlights the need for assessing treatment outcome in different settings to evaluate the effectiveness of the DOTS program. Treatment outcomes serve as proxy of the quality of TB treatment provided by the health care system, and identifying the plausible factors for unsuccessful treatment outcomes is vital to improve treatment approaches [28]. However, TB treatment outcomes and the underlying factors, to our knowledge, have not been studied in Ethiopian Somali regional state. Therefore, this study was aimed at assessing TB treatment success rate and factors associated with unsuccessful treatment outcomes in Ethiopian Somali region where more than 85% of the total population reside in rural areas and lead a pastoral way of life [29].

## Methods

### Study setting

**Fig. 1:**
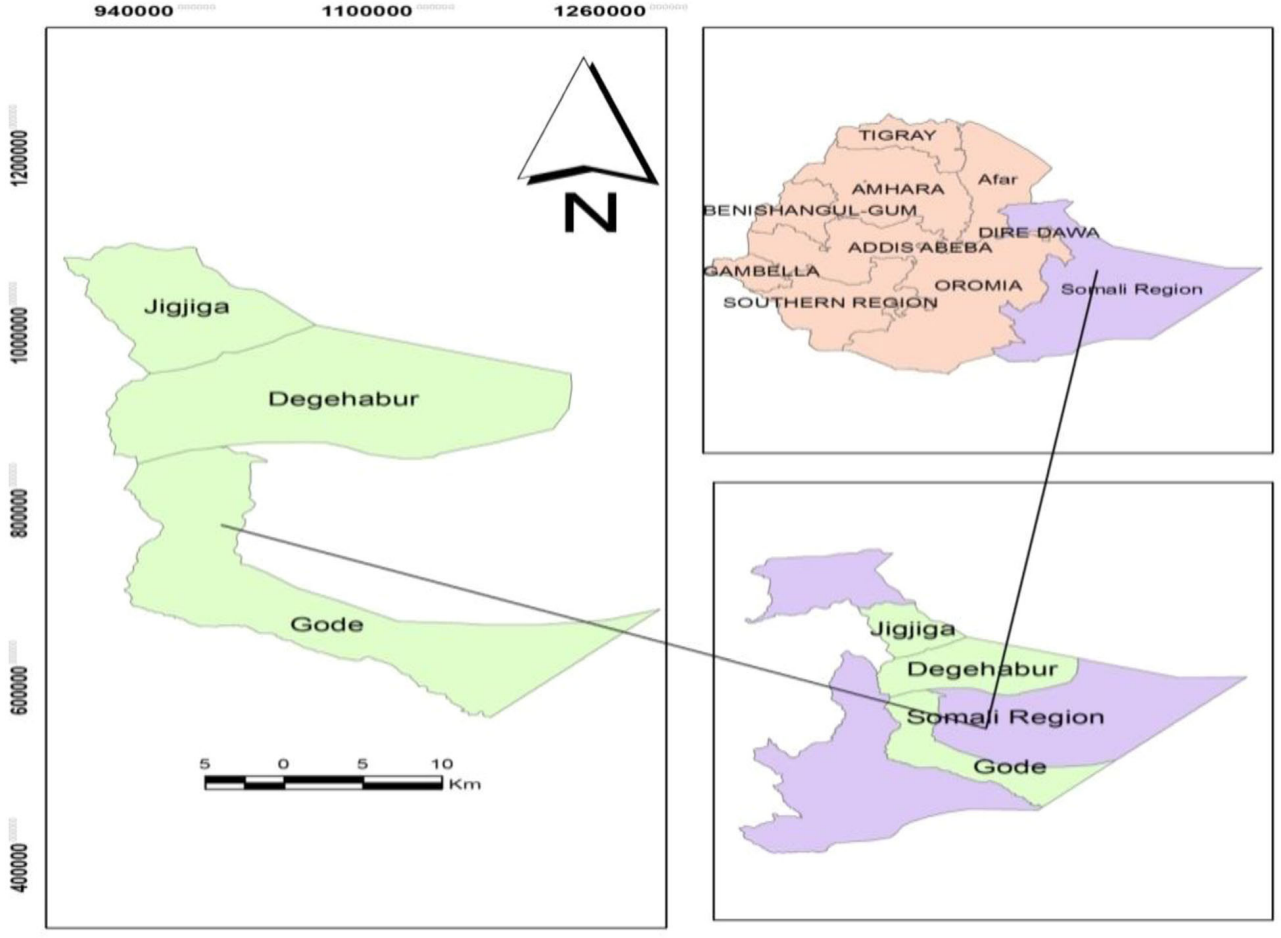
map of study area

The study was conducted in Kharamara, Dege-habour and Gode hospitals of Ethiopian Somali regional state. The hospitals were selected purposefully based on their higher patient load; both nomadic and agro-pastoralists inhabit in these areas, and township representation (Fig. 1). Kharamara hospital is located in the regional capital, Jigjiga, while Gode and Dege-habour are located in less urbanized zonal towns. TB clinics provide DOTS as per the National Tuberculosis Control Program guideline of Ethiopia. It involves taking a combination of drugs daily under direct supervision by a health care provider for 2 months (intensive phase), and then patients collect TB drugs to take home once a month for the remaining 4/6 months (continuation phase) [4].

### Study design and population

A retrospective chart review study was applied to retrospectively review the treatment outcomes of TB patients treated in the respective hospitals from September, 2009 to August, 2014. Patients with all forms of TB including PTB+, PTB- and EPTB were included. However, TB patients who were transferred out to other health facilities were excluded since their treatment outcome was unavailable unless the receiving health facility was tracked.

### Sample size and sampling

The minimum sample size calculated was 1173 using OpenEpi303 software for frequency in a population (proportion) [30]: assuming 95%CI, 50% hypothesized frequency of successful treatment outcome in the study area, 3% precision level and 10% missing rate. Systematic random sampling technique was employed to select the study participants from each hospital. Initially, we received the total number of TB patients treated in the three hospitals during the study period. The calculated sample size was divided to the three hospitals proportional to the number of TB patients treated in the respective hospitals. After the number of TB patients to be sampled from each hospital was determined, sampling intervals were calculated by dividing the total number of TB patients treated to the sample size needed in each hospital. Then the study participants were selected at sampling interval (unique for each hospital) starting from first to the last study year. The starting participants were selected randomly taking numbers from 1 to sampling interval. Finally, a total of 1378 patients with any form of TB was included.

### Data collection, processing and analysis

Data were extracted from patient record books using clinical chart abstraction sheet that contains the dependent variable, treatment outcome, and explanatory variables including age, sex, HIV status, smear type, treatment category and treatment year. Nurses working in other departments of the respective hospitals extracted the data after they were trained on the study objectives, sampling procedures and contents of record book. The data was checked for its completeness, consistency and reliability, and random rechecking was done on 5% of data prior to entry.

Data was double entered using EpiData 3.1 and exported to SPSS version 20 for cleaning and analysis. Descriptive statistics was performed to describe treatment success, explanatory variables and trends over 5 years period. Chi-square (χ^2^) test and logistic regression model were used to demonstrate the potential predictors of unsuccessful treatment outcome and statistical significance was determined at *P* ≤ 0.05. Variables with *P* ≤ 0.5 in the bivariate analysis were included in the multivariable analysis. Hosmer and Lemeshow method was used to check the fitness of logit model.

### Operational/standard definition of terms

- **Cure:** a pulmonary TB patient who was initially examined smear positive but examined smear negative after 5th and 6th months of treatment.
- **Successful treatment outcome**: patients who had completed and/or cured at the end of TB treatment regimen.
- **Unsuccessful treatment outcome**: patients who had defaulted from treatment or failed to cure at 5th month or died during the TB treatment regimen.
- **New case**: a patient who has never had treatment for TB before, or has been on anti-TB treatment less than 4 weeks.
- **Treatment failure**: a patient who, while on treatment, is smear-positive at the end of the 5th month or later, after commencing, or a patient who was initially sputum smear-negative but who becomes smear-positive during treatment regimen.
- **Default**: A patient who had initiated treatment but defaulted from treatment before completing the regimen.
- **Retreatment case**: a patient who had been treated for any form of TB before but has initiated treatment again following relapse or default or failure to cure of the 1st regimen.
- **Relapse**: a patient who has been declared cured or has completed treatment of any form of TB in the past but who reported back and was found to be smear positive.
- **Transfer In**: a Patient who started treatment in another health facility (reporting unit) and transferred to study hospitals (receiving units) to continue treatment.

## Result

### Socio-demographic and clinical characteristics of patients

A total of 1378 TB patients who were treated from September, 2009 to August, 2014 was included in the study. The median age was 26 years (range, 0 to 92 years), the majority of patients were male (59.1%) and new TB cases (90.6%), half of them (49.2%) were PTB-, and 4.6% were co-infected with HIV (Table 1 *)*.

**Table 1:** Socio-demographic and Clinical characteristics of study participants from three Hospitals, Ethiopian Somali Regional State

Characteristics		Treatment year					
		September, 2009 to August, 2010 *N* (%)	September, 2010 to August, 2011 *N* (%)	September, 2011 to August, 2012 *N* (%)	September, 2012 to August, 2013 *N* (%)	September, 2013 to August, 2014 *N* (%)	Total *N* (%)
Sex	Male	57 (60.6)	149 (61.6)	176 (57.9)	129 (54.9)	303 (60.2)	814 (59.1)
	Female	37 (39.4)	93 (38.4)	128 (42.1)	106 (45.1)	200 (39.8)	564 (40.9)
Age	≤ 14	19 (20.2)	39 (16.1)	60 (19.7)	40 (17.0)	84 (16.7)	242 (17.6)
	15 to 29	27 (28.7)	113 (46.7)	114 (37.5)	89 (37.9)	179 (35.6)	522 (37.9)
	30 to 44	30 (31.9)	49 (20.2)	66 (21.7)	50 (21.3)	100 (19.9)	295 (21.4)
	45 to 59	9 (9.6)	29 (12.0)	24 (7.9)	29 (12.3)	66 (13.1)	157 (11.4)
	60+	9 (9.6)	12 (5.0)	40 (13.2)	27 (11.5)	74 (14.7)	162 (11.8)
HIV Sero-status	Reactive	2 (2.1)	21 (8.8)	20 (6.6)	7 (3.0)	13 (2.6)	63 (4.6)
	Nonreactive	92 (97.9)	218 (91.2)	284 (93.4)	227 (97.0)	490 (97.4)	1311 (95.4)
Treatment category	New	80 (86.0)	201 (83.4)	274 (90.1)	213 (91.8)	475 (94.6)	1243 (90.6)
	Retreatment	8 (8.6)	24 (10.0)	26 (8.6)	7 (3.0)	13 (2.6)	78 (5.7)
	Transfer in	5 (5.4)	16 (6.6)	4 (1.3)	12 (5.2)	14 (2.8)	51 (3.7)
TB type	PTB+	25 (26.6)	50 (20.7)	84 (27.6)	67 (28.6)	118 (23.5)	344 (25.0)
	PTB-	51 (54.3)	125 (51.7)	139 (45.7)	107 (45.7)	255 (50.8)	677 (49.2)
	EPTB	18 (19.1)	67 (27.7)	81 (26.6)	60 (25.6)	129 (25.7)	355 (25.8)
Total per year		94 (6.8)	242 (17.6)	304 (22.1)	235 (17.1)	503 (36.5)	1378 (100)

*PTB+* Pulmonary Tuberculosis Smear Positive, *PTB* Pulmonary Tuberculosis Smear Negative, *EPTB* Extra-Pulmonary Tuberculosis

### Treatment success and its trend

Of the total 1378 TB patients, 16 (1.2%) had missed data on treatment outcome. The overall treatment success rate was 86.8% [95%CI = 84.9%–88.5%] for all forms of TB across the 5 years. The overall death and default rates were 4.8% and 7.6% respectively. Majority (60.6%) of died patients were between 18 to 65 years old. Of those with unsuccessful treatment outcomes, 57.8%, 36.7% and 5.6% were defaulters, dead and failures respectively (Table 2).

**Table 2:** Trend of treatment outcomes of TB patients from three hospitals, Ethiopian Somali Regional State

Treatment Outcome (*n* = 1362)	Treatment year					
	September, 2009 to August, 2010 *n* (%)	September, 2010 to August, 2011 *n* (%)	September, 2011 to August, 2012 *n* (%)	September, 2012 to August, 2013 *n* (%)	September, 2013 to August, 2014 *n* (%)	Total *n* (%)
Cured	18 (19.2)	37 (15.4)	64 (21.4)	53 (22.6)	90 (18.2)	262 (19.3)
Completed	68 (72.3)	180 (75.0)	217 (72.6)	165 (70.2)	290 (58.7)	920 (67.5)
Total successful	86 (91.5)	217 (90.4)	281 (94.0)	218 (92.8)	380 (76.9)	1182 (86.8)
Died	5 (5.3)	16 (6.7)	8 (2.7)	12 (5.1)	25 (5.1)	66 (4.8)
Failure	1 (1.1)	1 (0.4)	4 (1.3)	1 (0.4)	3 (0.6)	10 (0.7)
Defaulted	2 (2.1)	6 (2.5)	6 (2.0)	4 (1.7)	86 (17.4)	104 (7.6)
Total unsuccessful	8 (8.5)	23 (9.6)	18 (6.0)	17 (7.2)	114 (23.1)	180(13.2)

**Fig. 2:**
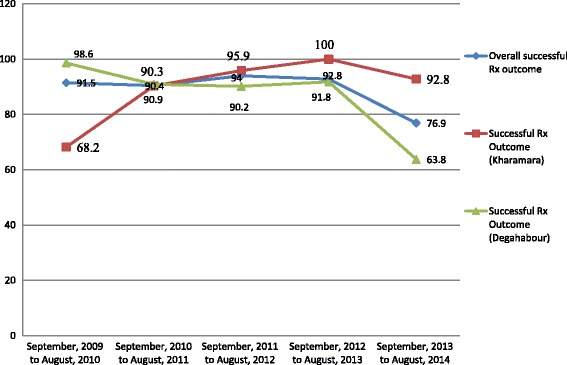
Trend of successful TB treatment outcome in three hospital of Ethiopian Somali Regional State, September 2009 to August 2014

The trend of treatment success rates showed fluctuating drift. It was above 90% in the years from September 2009 to August 2013 but declined to 76.9% from September 2013 to August 2014.^1^ The overall treatment success rates were 82.9%, 83.7% and 92.7% at Degehabour, Gode and Kharamara Hospitals respectively. A default rate of 28.5% (*n* = 59) was observed at Degehabour hospital from September 2013 to August 2014 when the success rate was 63.8% (Fig. 2).

**Fig. 3:**
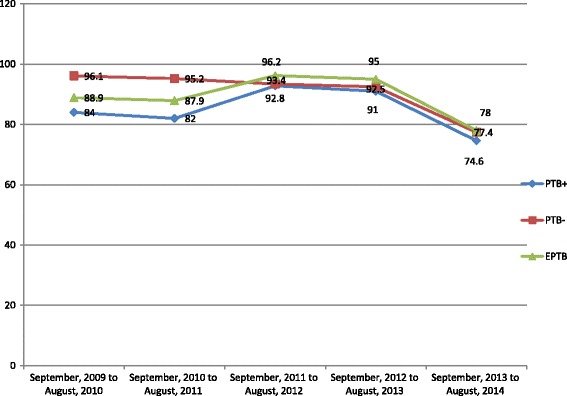
Trend of successful TB treatment outcome by TB type in three hospitals of Ethiopian Somali Regional State, September 2009 to August 2014

The treatment success rates were 84%, 87.8% and 87.6% among PTB+, PTB- and EPTB patients respectively. The trend of treatment success rate of PTB- patients showed a continuous decrease from 96.1% down to 77.4%. However, it had fluctuating trends among PTB+ and EPTB patients and the bottommost success rates of 78% and 74.6% were observed respectively (Fig. 3).

### Predictors associated with unsuccessful treatment outcome

The overall treatment success rate was similar between males (86.1%) and females (87.8%) [*p* = 0.6], and between PTB+ (84.0%), PTB- (87.8%) and EPTB (87.6%) patients [*p* = 0.2]. The adjusted multivariable logistic regression revealed that the odds of unsuccessful treatment outcome was considerably higher among re-treatment TB patients [AOR =2.4; 95%CI =1.4–3.9]; patients treated from September 2013 to August 2014 [AOR = 3.6; 95%CI = 1.7–7.8]; and those treated in less urbanized hospitals [*P* < 0.001] compared to their respective counterparts (Table 3). The higher odds of unsuccessful outcome observed among retreatment TB patients (Table 3) was due to higher death (10.2%) and failure to cure (4.7%) rates compared to new counterparts (Table 4). Pulmonary TB smear positivity was higher among retreatment (53.8%) patients compared to new (22.8%) counterparts [AOR = 2.3; 95% CI = 1.6–3.5; *p* < 0.001].

**Table 3:** Predictors of unsuccessful treatment outcome of TB patients from three hospitals, Ethiopian Somali Regional state

Characteristics		Total n (%) of TB Cases	Unsuccessful outcome *n* (%)	*p*-value	COR (95% CI)	*P*-value	AOR (95% CI)
Sex	Male	804 (59.0)	112 (13.9)	0.35	1.16 (0.85, 1.61)	0.6	1.1 (0.8, 1.6)
	Female	558 (41.0)	68 (12.2)		1		1
Age group	≤ 18	367 (26.9)	44 (12.0)	0.8^a^	1		1
	19 to 26	326 (23.9)	43 (13.2)		1.1 (0.7, 1.7)		
	27 to 40	329 (24.2)	43 (13.1)		1.1 (0.7, 1.7)		
	41+	340 (25.0)	50 (14.7)		1.3 (0.8, 2.0)		
HIV Sero-status	Reactive	63 (4.6)	10 (15.9)	0.5	1.2 (0.6, 2.5)	0.4	1.4 (0.7, 3.0)
	Non-reactive (reference)	1295 (95.4)	170 (13.1)		1		1
Treatment Category	New (reference	1228 (90.6)	151 (12.3)	0.006	1	0.001	1
	Re-treatment	128 (9.4)	27 (21.1)		1.9 (1.2, 3.0)		2.4 (1.4, 3.9)
TB type	PTB+	343 (25.2)	55 (16.0)	0.2	1.4 (0.9, 2.1)	0.2	1.3 (0.8, 2.0)
	PTB-	671 (49.3)	82 (12.2)		0.9 (0.7, 1.5)		0.9 (0.6, 1.3)
	EPTB (reference)	346 (25.4)	43 (12.4)		1		
Treatment year	Sept2009 - Aug2010 (refernce)	94 (6.9)	8 (8.5)	<0.001	1	< 0.001	1
	Sept2010 - Aug2011	240 (17.6)	23 (9.6)		1.1 (0.5, 2.6)		1.1 (0.5, 2.6)
	Sept2011 - Aug2012	299 (22.0)	18 (6.0)		0.7 (0.3, 1.6)		0.7 (0.3, 1.7)
	Sept2012 - Aug2013	235 (17.3)	17 (7.2)		0.8 (0.3, 2.0)		0.8 (0.3, 1.9)
	Sept2013 - Aug2014	494 (36.3)	114 (23.1)		3.2 (1.5, 6.9)		3.6 (1.7, 7.8)
Hospital	Kharamara	522(38.3)	38(7.3)	<0.001	1	<0.001	1
	Gode	227(16.7)	37(16.3)		2.5(1.5, 4.0)		3.1(1.8, 5.1)
	Dege-Habour	613(45)	105(17.1)		2.6(1.8, 3.9)		3.0(2.0, 4.6)

^a^Indicates the variable not included in multivariable logistic regression, *PTB+* Pulmonary Tuberculosis Smear Positive, *PTB* Pulmonary Tuberculosis Smear Negative, *EPTB* Extra-Pulmonary Tuberculosis

**Table 4:** Treatment outcomes of new and retreatment TB patients from three hospitals, Ethiopian Somali Regional state

Treatment Category	Treatment outcome			
	Successful *n* (%)	Died *n* (%)	Failure *n* (%)	Defaulted *n* (%)
New (*n* = 1228)	1077 (87.7)	52 (4.2)	4 (0.3)	95 (7.7)
Retreatment (*n* = 128)	101 (78.9)	13 (10.2)	6 (4.7)	8 (6.2)

## Discussion

The majority of TB patients were male and new TB cases, and nearly half were PTB-. It is similar to reports from Southern [21] and Northwest Ethiopia [22]. It is evidenced that female TB patients in developing countries did not seek timely care compared to males [31–35]. Therefore, this higher proportion of male patients could be due to better utilization of TB services or higher proportion of the disease compared to females. On the other hand, the HIV co-infection (4.6%) is relatively lower compared to 12.7% in Debre Tabor [22], 26.1% in Bahir Dar [27] and 38.1% in Azezo [26] areas, Northwest Ethiopia; 10% in Arsi Negele, Southern Ethiopia [20]; and 8.6% in Tigray region, Northern Ethiopia [19]. This is probably related to the low general HIV prevalence in pastoralist regions of Ethiopia unlike the agrarian regions [36].

The 86.8% treatment success rate for all forms of TB was nearly comparable to the WHO 2015 report for Ethiopia (89%), High-Burden Countries (88%) and globe-wise respectively (86%) [1]. In addition, this agrees with varies studies such as 85.5% in Nigeria [37], 87.1% in Debre Tabor [22], 87.3% in Arsi Negele [20], 87.8% in Dabat [23] and 89.2% in Tigray [19] areas of Ethiopia. In contrast to these, our finding is notably high compared to 46.2% in Nigeria [38], 67.3% in Malaysia [14], 74.4% in South Africa [39], and 80.5% in Azezo [26] and 80.8% in Bahir Dar [27] towns of Ethiopia. The discrepancies may be attributable to variations in study setting, population and duration, sample size, and HIV prevalence. HIV has been identified as main factor of poor treatment outcomes [24, 26, 27]. Moreover, the performance of health facilities and health systems in implementing DOTs might differ.

Death (4.8%) and default (7.6%) rates contributed much of the unsuccessful treatment outcomes and nearly two-third of died patients were productive age people (18 to 65 years old). The death rate is comparable to 5.6% in Debre Tabor, 3.9% in Tigray, 3.6% in Arsi Negele and 3.1% in Dabat areas of Ethiopia [19, 20, 22, 23]. On contrary, it is lesser compared to 14.6% in Bahir Dar, Ethiopia [27], 9.5% and 11.5% in Nigeria [37, 38], 9.8% in South Africa [39] and 17.6% in Malaysia [14]. However, the actual TB provoked death would have been even lower since the actual causes of death was not recorded, either caused by TB or other causes of death.

Despite the achievement of satisfactory treatment success, it was not steady over the 5 years rather showed fluctuating trend. It dropped from >90% in the first four study years down to 76.9% in the last study year (September 2013 to August 2014) when the highest default rate (17.4%) occurred. This indicates that successful treatment outcomes are not for granted. Achievement of rewarding treatment outcomes in a certain year and health facility is not warranty of persistence successes. This is, therefore, a signal for the health care system that there should be incessant efforts consistently in all the treatment delivery points. Other studies pointed out similar findings [13, 26, 40].

Treatment category, treatment year and hospital had significantly associations with unsuccessful treatment outcome [*P* < 0.05]. Nevertheless, no significant associations were observed between unsuccessful outcome and gender, age, HIV sero-status and TB type [*P* > 0.05] unlike other studies elsewhere [16, 18, 20, 38, 39]. Retreatment TB patients experienced higher rate of death (10.2%)/failure to cure (4.7%) compared to new TB patients [AOR = 2.4; 95%CI = 1.4–3.9]. This is supported by varies studies [19, 27, 41, 42]. Retreatment patients had history of treatment in the past but the treatment was initiated again following relapse or default or failure to cure of the past treatment. The unsuccessful treatment outcome in the past might lead to the development of hazardous or drug resistant strains [11]. Therefore, the retreatment cases would be infected by drug resistant strains acquired during the preceding treatment.

Most importantly, more than half (53.8%) of retreatment patients were PTB+ at diagnosis compared to 22.8% among new patients [*p* < 0.001]. PTB+ patients serve as sources of infection transmission to close contacts in households and congregate settings [43, 44]. This implies that retreatment patients can be sources of infections by hazardous or drug resistant strains, and their lesser tendency to cure prolongs the infectious period. This may, therefore, be an alarm to consider special treatment response follow-up mechanisms for retreatment cases beyond the standard regimen in order to prevent adverse outcomes and interrupt the transmissions.

The trend of treatment success rate was not linear over the years but the likelihood of unsuccessful outcome was threefold elevated (23.1%) during the last study year (September 2013 to August 2014) compared to first study year (8.5%). This was attributable to higher default rate (28.5%) at Zonal hospital in less urbanized setting.

Treatment success rates varied among hospitals [*p* < 0.001]. The referral hospital in the regional state’s capital achieved highest (92.7%) success rate compared to the two other hospitals (83.7% and 82.9%) which had relatively higher default rates and located in less urbanized zonal towns. This discrepancy could be related to patients’ compliance to treatment or healthcare providers’ performance. Majority of the people in lower performing hospitals are pastoralist and the recurrent droughts usually force pastoralists to migrate for pasture. In addition, these hospitals usually suffer higher health worker turnover as they are located in less urbanized towns with poor access to infrastructure and harshly weather. This inter-facility variation is supported by studies in Nigeria [37, 38], Northwest Ethiopia [23, 26] and Southern Ethiopia [20, 45]. However, we could not examine the difference between urban and rural patients since patient addresses were not well documented in the register.

Our study is subjected to some limitations. We were not able to assess all predictors of unsuccessful TB treatment outcome since the patient register book did not have records of some important variables including residence, income, family size, occupation, and the presence of other co-morbidities that could affect treatment outcomes. The real cause of death was not recorded which would have helped to rule out deaths by other causes. Furthermore, we could not find data on daily adherence of patients to their treatment that would have influenced their treatment outcomes.

## Conclusion

The DOTS strategy seems to have enhanced TB treatment success in Ethiopian Somali Regional State where the overall treatment success rate for all forms of TB aligned to the WHO target for 2011–2015. However, the trend of treatment success was not linear, rather unpredictably adverse treatment outcomes could occur anywhere and anytime. Moreover, TB patients with previous history of treatment had higher odds of death or failure to cure after initiation of retreatment. Forefront of the higher risk of unsuccessful treatment outcome, the majority of retreatment patients were PTB+ cases so that it could enhance the transmission of vastly hazardous strains to susceptible persons. Hence, continual effort to effectively execute DOTS strategy should be strengthened universally in all health facilities as undesirable outcomes can occur intermittently. What’s more, DOTS providers should monitor retreatment TB cases more frequently than the recommended interval to evaluate their response to retreatment and take early actions accordingly. Further prospective studies with strong design could help to revise the current follow-up guideline.

## Abbreviations

AOR: Adjusted odds ratio
CI: Confidence interval
COR: Crude odds ratio
DOTS: Directly observed therapy-short course
EPTB: Extra-pulmonary tuberculosis
HIV: Human immuno-deficiency virus
IQR: Inter-quartile range
PTB: Pulmonary tuberculosis smear negative
PTB +: Pulmonary tuberculosis smear positive
RHZES: Rifampicin, isoniazid, pyrazinamide, ethambutol and streptomycin
TB: Tuberculosis

## Footnotes

1 Ethiopian calendar begins in September and ends in August so that the years written ‘from September to August” in this document indicate Ethiopian fiscal years.
